# Supplementary material for: Retrospective model-based inference guides model-free credit assignment
Source: Nat Commun. 2019 Feb 14;10:750. doi: 10.1038/s41467-019-08662-8 (PMC6375980; doi:10.1038/s41467-019-08662-8)
Supplement: Supplementary file 1 — Supplementary Information [file 41467_2019_8662_MOESM1_ESM.pdf]

Supplementary Information

Retrospective Model-Based inference Guides Model-Free Credit Assignment

Moran et al.

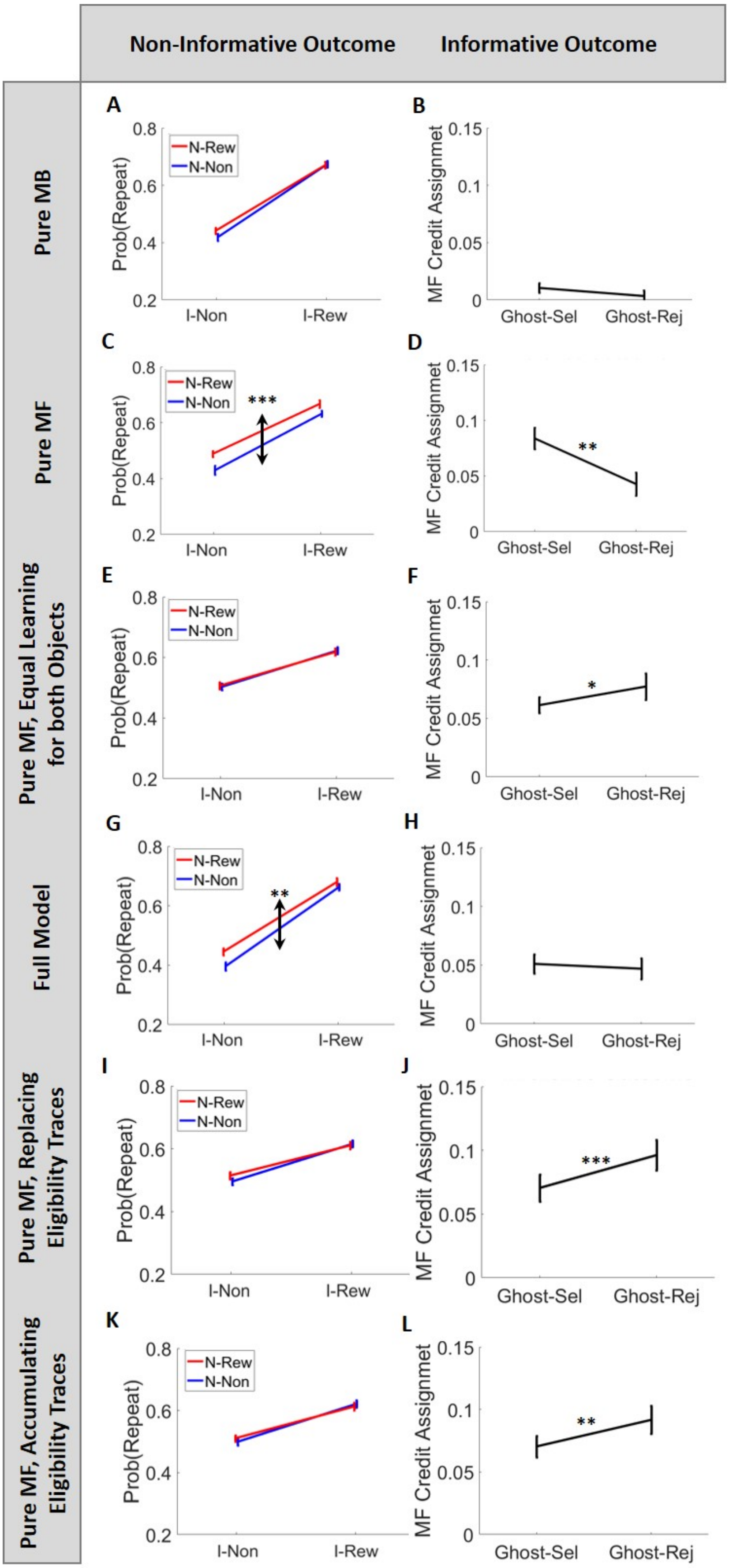
Non-Informative Outcome
Informative Outcome
Pure MB
Pure MF
Pure MF, Equal Learning for both Objects
Full Model
Pure MF, Replacing Eligibility Traces
Pure MF, Accumulating Eligibility Traces
A
B
C
D
E
F
G
H
I
J
K
L
Prob(Repeat)
MF Credit Assignmet
N-Rew
N-Non
I-Non
I-Rew
Ghost-Sel
Ghost-Rej

Supplementary Figure 1. Related to Fig. 4. Predictions of different models with respect to analyses that show preferential MF credit assignment for the ghost-nominated object presented in Fig. 4C-D. (A-B) A pure MB model predicted neither the main effect of the Common-outcome on repetition probability (Fig. 4C) neither the “ghost MF credit-assignment contrast” (Fig. 4D). (C-D) A pure MF action model, on the other hand, predicted both a positive Common-room effect and a positive contrast. (E-F) However, when we simulated once more the pure MF model but imposing equality between the learning rates for the ghost-nominated and ghost-rejected objects (both were set to their mean value) the model predicted no Common-outcome effect and a negative, unlike the empirical positive, contrast. (G-H) The full model predicted a positive Common-outcome effect but was unable to predict the contrast. (I-J) A pure MF model with replacing eligibility traces predicted no Common-outcome effect and a negative ghost MF credit-assignment contrast. (K-L) A pure MF model with accumulating eligibility traces predicted no Common-outcome effect and a negative ghost MF credit-assignment contrast and. These model simulations show that a higher learning rate for the ghost-nominated as compared to the ghost rejected object, as in the pure MF model, seems necessary to generate a positive Common-outcome effect on choice repetition and a positive ghost MF credit-assignment contrast. The notation in the figure is like in Fig. 4. Error bars correspond to SEM across participants calculated separately in each condition (n=40). See Supplementary Figure 3 for more simulations using the object-value learning MB formulation.

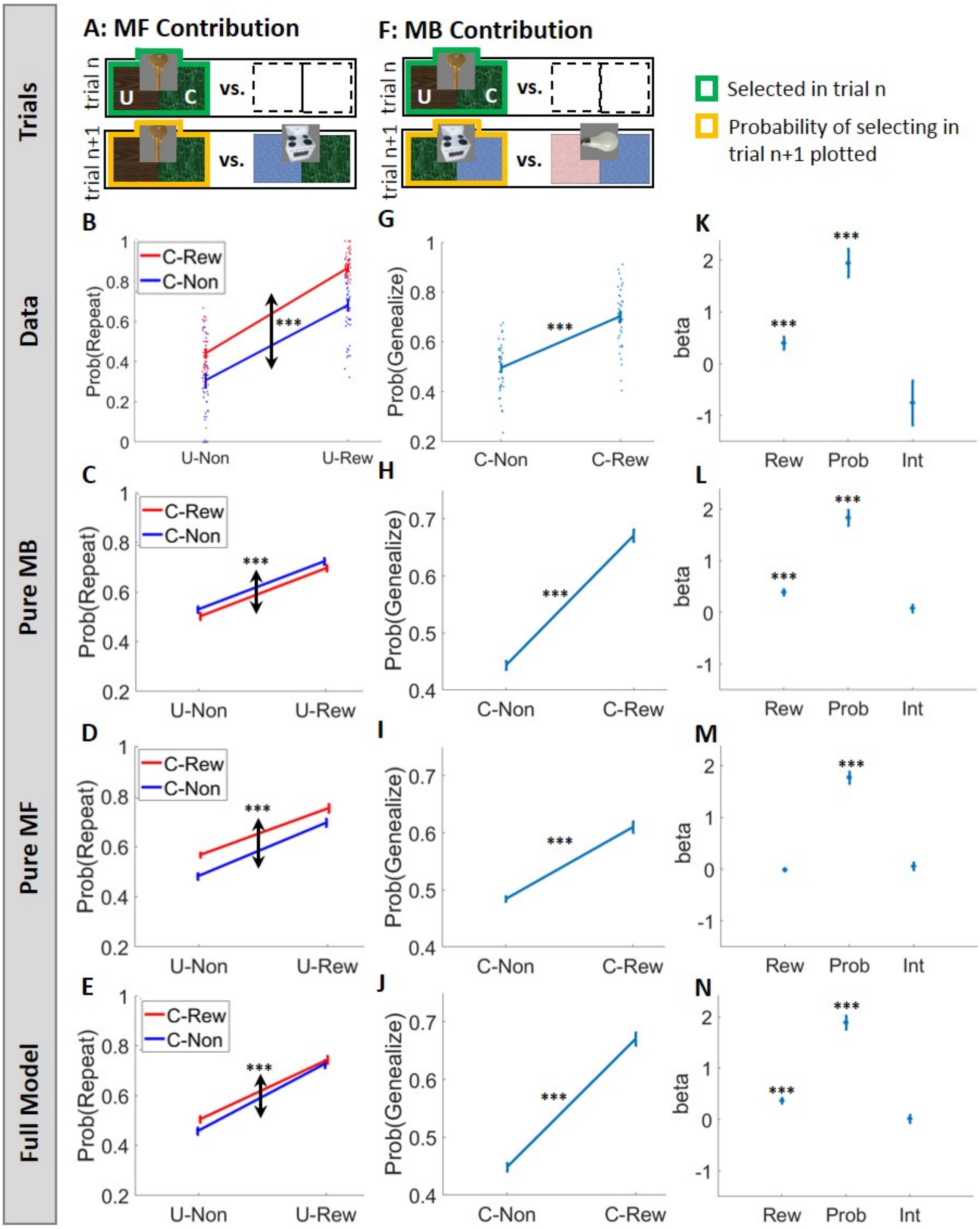
Trials
Data
Pure MB
Pure MF
Full Model
A: MF Contribution
F: MB Contribution
trial n
trial n+1
U
C
vs.
Selected in trial n
Probability of selecting in trial n+1 plotted
B
C
D
E
G
H
I
J
K
L
M
N
C-Rew
C-Non
Prob(Repeat)
Prob(Genealize)
U-Non
U-Rew
C-Non
C-Rew
beta
Rew
Prob
Int
***

Supplementary Figure 2. MF and MB contributions to performance based on the *object-value learning* formulation. Related to Fig 2. (A-B) Repeated from Fig. 2A for convenience. (C) A pure MB model predicted a negative, unlike the empirical positive, Common room main effect. (D) Note that the object-value learning formulation differed from the room-value learning formulation only with respect to MB but not MF. Hence this panel is the same as Fig 2D. (E) The full model predicted a positive Common-outcome main effect. (F-G, K) Repeated for convenience from Fig. 2F-G, K. (H, L) A pure MB predicted a positive Common-reward regression coefficient. (I, M) Repeated for convenience from Fig. 2I, M. (J, N) The full model also predicted a positive coefficient for the Common outcome. The notation is the same as Fig. 2. Error bars correspond to SEM across participants calculated separately in each condition (n=40). Images adapted from the stimulus set of Kiani et al. 2007, Ref. 41.

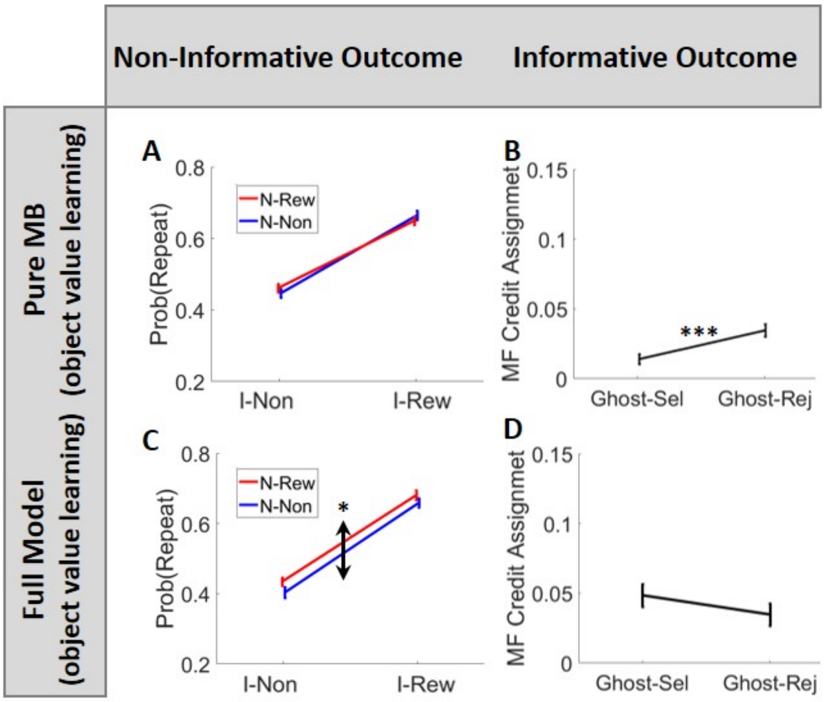

Supplementary Figure 3. Related to Fig. 4. Similar to Supplementary Figure 1 but for the object-value learning MB formulation. (A-B) A pure MB model failed to predict the main effect of the Common-outcome on repetition probability and predicted a negative, unlike the empirical positive, “ghost MF credit-assignment” contrast. (C-D) The full model predicted the positive Common-room effect and was unable to predict a significant contrast. Because object-value learning models differed from the room-value learning models only with respect to MB but not MF, no panels in the current figure correspond to Supplementary Figure 1 (E-L). The notation is the same as in Fig. 4. Error bars correspond to SEM across participants calculated separately in each condition (n=40).

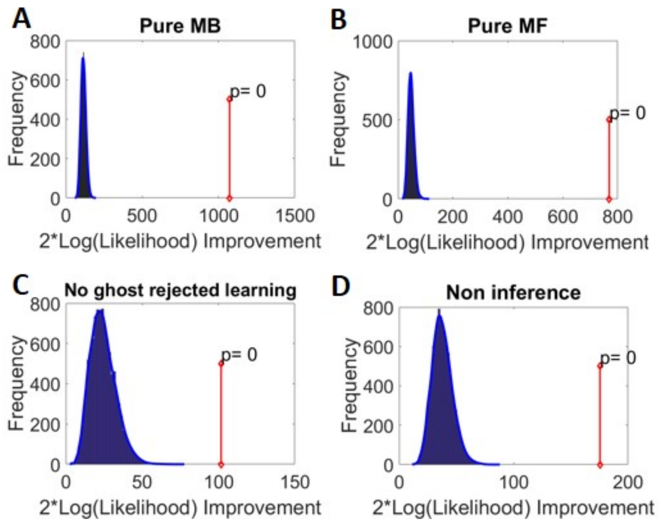

Supplementary Figure 4. Related to Fig. 5. Model-comparison, BGLRT, results for the *object-learning value* family of models. The structure of the figure is the same as Fig. 5. As for the room-value learning family, the full model was rejected at the group level, when compared to each of the 4 sub-models (all p<.001). The individual models were rejected at p=.05 for 33 (pure MB), 32 participants (pure MF-action), 9 (no ghost-rejected learning) and 14 (no-inference) participants, respectively.

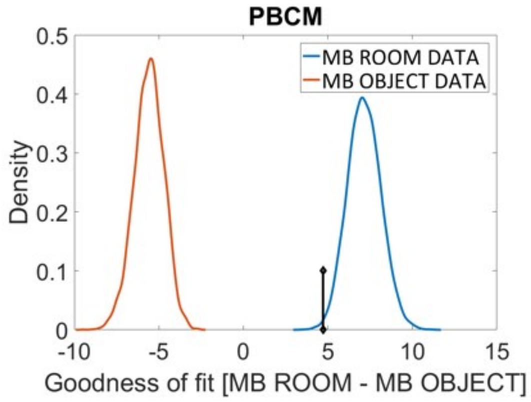

Supplementary Figure 5. Related to Fig. 4 and Supplementary Figure 4. A PBCM comparison (see methods) for the room-value learning and the object-value learning full models. The abscissa displays the difference (room-value learning minus object-value learning) in twice the log maximal likelihood for ML fits, averaged across participants. When the room-value learning model generated synthetic data, it provided a higher average likelihood (blue distribution). Conversely, when the object-value learning model generated synthetic data, it provided a higher average likelihood (brown distribution). The empirical average likelihood difference (black) was positive, supporting the room-value learning as a better description for group performance. The room-value learning model also provided a higher maximal likelihood than for 29 participants.

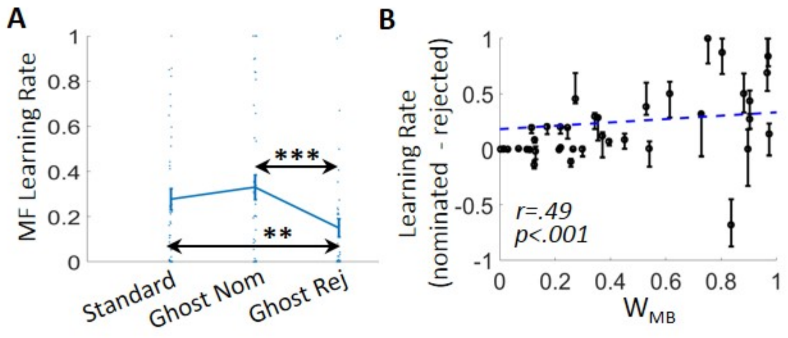

Supplementary Figure 6. Analyses based on the ML parameters of the *object-value learning* full model. The structure of this figure is identical to Fig. 6 and it supports the same conclusions. In panel A Error bars correspond to SEM across participants calculated separately in each condition (n=40). In panel B error bars represent the densest interval that contained 50% of the mass of the estimated learning-rate difference distribution obtained by parametrically bootstrapping the data (see methods for details)

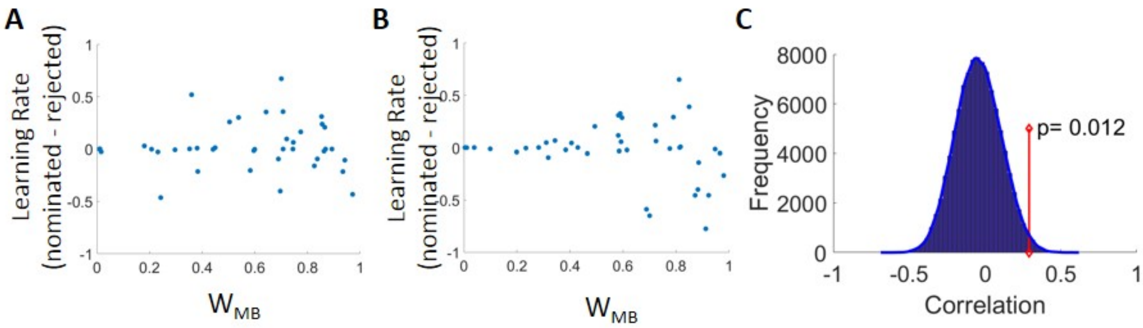

Supplementary Figure 7. Related to Fig. 6B. A bootstrap permutation test for the correlation between model-baseness and preferential MF learning for the ghost-nominated object (see methods). (A-B), two example scatter plots as in Fig 6B, that were obtained by fitting the full model to data that was parametrically bootstrapped from the non-inference model. (C) A null distribution for the no-correlation hypothesis (blue). The data (red) supported a significant correlation.

Supplementary Note 1. Related to Fig. 2 and 4. The purpose of this note is to help readers interpret figures 2 and 4. We use Fig. 2A-B as a working example, but similar considerations apply for the other panels.

One striking aspects of Fig 2B is that the repetition probability is <0.5 when the Common room is rewarded and the Unique room is not, but >0.5 in the case that the Unique room is rewarded and the Common room is not. This occurs despite the fact that the effects of both the Common and Unique rooms were significantly positive in the logistic model. We explain how these empirical patterns are consistent with the results of the logistic regression. First, the positive effect of the common room reflects the fact that the C-rew (red) line is above the C-non (blue) line for both values of the unique outcome (U-non and U-rew). However, the predicted probabilities from the regression model are influenced by all regression coefficients: the intercept, the effects of the common and unique rooms and by their interaction. These probabilities cannot be compared against a nominal baseline probability of 0.5, as we detail below.

For a worked example: in the relevant regression model, coding reward and non-reward as +0.5 and -0.5 respectively, the coefficients were:

beta coefficient for:	value
intercept	0.41
common	0.98
unique	2.03
common_unique_interaction	0.46

Thus, the predicted repetition probability (in logistic units) for the case for which the common room is rewarded and the unique room is unrewarded is
beta_intercept + 0.5*beta_common - 0.5*beta_unique - 0.25*beta_interaction= -0.23.
That this value is negative corresponds to a nominal repetition probability lower than 0.5. The repetition-probability for the mirror case for which the common room is unrewarded but the unique room is rewarded can be similarly calculated as
beta_intercept - 0.5*beta_common + 0.5*beta_unique - 0.25*beta_interaction= 0.82
and thus a repetition probability which is > .5.
Thus, the regression model is able to predict probabilities that were higher and lower than .5, in these two cases, respectively, for the very reason that beta_unique> beta_common.

Another confusing issue is whether the <0.5 repetition probability (Fig. 2B) in the case of a Common room reward and Unique non-reward is consistent with a MF contribution to choice. Importantly, the data in the focal figure do not reflect a pure MF contribution. Rather, the empirical data reflect the non-linear aggregation of a multitude of influences including, but not limited to, the MF contribution. These additional influences are:
1) A contribution from a MB system,
2) A tendency for perseveration (which could be either positive or negative) and

3) The historical values of the objects as determined by past reward events that occurred before trial n.
The impact of these terms implies that a comparison should not be made with a probability of 0.5 to test whether or not there are MF contributions to behaviour. To elaborate, consider the predictions of a pure MB agent: when the common room is rewarded and the uncommon room is unrewarded, a pure MB agent can predict a p<0.5 repetition probability. The reason is that the common reward is “cancelled out” from MB calculations on trial n+1, whereas the unique non-reward decreases the value of the repetition object relative to the object that is the alternative on trial n+1. In support of this argument, when we simulated the behaviour of a pure MB agent based on the best-fitting parameters of our full model but knocked out contributions from both the MF system (setting w_{MB}=1) and perseveration (setting Perseveration=0) we found that the model predicted a repetition probability <0.5. In sum, the repetition probability reflects the aggregation of several influences, some acting to increase it but others acting to decrease it. The predicted probability will therefore depend on the specific parameters that determine how these influences combine. Critically, a repetition probability < 0.5 is consistent with contributions from a MF system.

Finally, readers may wonder whether the stronger effect of the Unique reward (as compared to the Common reward) is a marker for a MB contribution to choices. The somewhat surprising answer is negative. A pure MF-action system can also predict a higher influence for the unique than the common room. This is evident, for example, in Fig. 2D, in which MB doesn’t contribute directly to choices. Therefore, a larger unique influence does not implicate the contributions of a MB system.

Parameter	w_{MB}	β	p	$\mathrm{lr}_{\mathrm{MB}}$	$\mathrm{lr}_{\mathrm{standard}}$	$\mathrm{lr}_{\text{ghost-nom}}$	$\mathrm{lr}_{\text{ghost-rej}}$
25%	0.25	2.34	0.00	0.11	0.03	0.01	0.00
50%	0.53	4.17	0.03	0.36	0.24	0.33	0.08
75%	0.80	10.45	0.13	0.74	0.64	0.80	0.38

Supplementary Table 1. Best fitting parameters for the full RL model. The table shows the 25, 50 and 75 percentiles across participants. w_{MB} is the relative contribution of the MB system to choices; β is the inverse temperature governing choices; p is the perseverance towards the previously executed bandit; $\mathrm{lr}_{\mathrm{MB}}$ is the learning rate for the MB system; $\mathrm{lr}_{\mathrm{standard}}$ is the MF learning rate for standard trials; $\mathrm{lr}_{\text{ghost-nom}}$ is the MF learning rate for the ghost-nominated (retrospectively-inferred) object on uncertainty trials; $\mathrm{lr}_{\text{ghost-rej}}$ is the MF learning rate for the ghost-rejected object on uncertainty trial. See methods for a complete description of the model and its parameters.
